# “Virginity” and the hymen: knowledge, attitudes, and practices among Swiss health professionals

**DOI:** 10.1093/sexmed/qfag071

**Published:** 2026-08-06

**Authors:** Pace Margherita, Yaron Michal, Abdulcadir Jasmine

**Affiliations:** Department of Pediatrics, Obstetrics and Gynaecology, Geneva University Hospitals, Geneva, 1211, Switzerland; Department of Pediatrics, Obstetrics and Gynaecology, Geneva University Hospitals, Geneva, 1211, Switzerland; Department of Pediatrics, Obstetrics and Gynaecology, Geneva University Hospitals, Geneva, 1211, Switzerland

**Keywords:** hymen, hymenoplasty, virginity, female genital mutilation

## Abstract

**Introduction:**

Hymen anatomy and physiology remain poorly understood, even among health-care providers. Misconceptions about virginity testing, virginity certification, and hymenoplasty can lead to harmful practices and place women who are unable to prove their virginity at risk of violence or even death. The aim of this study is to investigate the knowledge, attitudes, and practices of gynecologists and pediatricians in Switzerland regarding virginity testing, virginity certification, and hymenoplasty, and to identify gaps to inform future training and professional guidelines.

**Methods:**

Gynecologists, pediatricians, and medical students completed a 22-question survey. Group differences were assessed using chi-square or Fisher’s exact tests, with significance set at 5%.

**Results:**

A total of 542 participants completed the survey. Overall, 97.5% believed that the absence of bleeding during the first vaginal penetration does not mean that someone is not a “virgin.” However, for 13.3% of respondents, hymenal rupture does define the loss of virginity, and 24.5% believed that a gynecologist can determine virginity status, with a significant association with medical practice settings (*P* < .001), university hospitals (*P* = .01), private clinics (*P* = .009), and public institutions (*P* < .001). Hymenoplasty was considered effective by 16.2% of the participants in ensuring bleeding at subsequent penetration. The right of patients or family to request a virginity check was acknowledged by 18.9% of participants, even if most respondents (85.9%) believed that practicing hymenal reconstruction reinforces gender inequalities. In addition, 68% of participants agreed that a virginity certificate should be issued in life-threatening situations.

A little over one-third (39.3%) of the responding physicians had received at least one request for virginity testing, certification, or hymenal reconstructive surgery; 30.4% of them accepted the request, but only 28.8% of all requests offered multidisciplinary management.

**Discussion:**

Women may encounter different approaches depending on the health-care professional they consult, reflecting a lack of standardized care. This national survey highlights substantial knowledge gaps and variability in practice among Swiss health-care professionals, underscores the absence of official guidelines and a coordinated support network, and calls on Swiss medical societies to establish clear, evidence-based recommendations that prioritize patient information, safety, and support while integrating these topics into education, residency training, and medical school curricula.

## Introduction

The hymen is a membranous structure located at the entrance of the vaginal canal with no established biological function. Nevertheless, throughout history and across diverse cultural contexts, it has acquired considerable symbolic and social significance. Persistent myths include the assumption that hymenal morphology can indicate a woman’s sexual history or “virginity” status and the belief that the first vaginal penetration must result in bleeding—leading to the conviction that the absence of bleeding is evidence of prior sexual penetrative activity.^1^

In some cultures, the presumed “loss of virginity” can be considered a source of dishonor for women and their families, potentially jeopardizing marriage arrangements and, in extreme cases, resulting in rejection or even killing of the bride in an honour killing.^1^ In some instances, the lack of virginity may also hinder access to employment opportunities.^2^ In certain legal and cultural settings, the presence or absence of virginity is invoked as evidence in cases of alleged rape.^3^^,^^4^ In order to ensure, preserve, or “restore” virginity, several harmful practices are performed, such as female genital mutilation (FGM),^5^ hymenoplasty,^6^ and others, such as the Rbat and the Shilshalo.^7^^,^^8^ Furthermore, premarital virginity is often seen as reassuring men about paternity.^9^

Despite persistent beliefs, numerous studies have shown that hymenal appearance is not a reliable determinant of sexual activity or virginity.^2^^,^^10–17^ Research has also highlighted limited knowledge among health professionals regarding hymenal anatomy and function,^2^ as most pre- and postgraduate health-care curricula include little or no specific training on the topic. As a result, misconceptions remain widespread even among physicians, including gynecologists and pediatricians, who are most frequently confronted with such questions. These misconceptions perpetuate false beliefs and may contribute to harmful practices worldwide, such as virginity testing, which is associated with well-documented short- and long-term physical and psychosocial consequences.^4^

In 2020, virginity testing and certification were banned in France by specific legislation, following a 2018 WHO statement and a recommendation from the national medical society in the same year.^18^^,^^19^ Since virginity cannot be determined through physical examination, like any other form of false medical certification, both virginity testing and virginity certification are inherently invalid and unlawful. To support gynecologists faced with such requests, the national medical society issued an informational document for patients, including a list of organizations and associations qualified to provide assistance—particularly women’s rights groups and organizations dedicated to supporting individuals exposed to violence.^20^ The law has generated extensive debate among French gynecologists. The most frequently reported criticism concerns its limited practical impact: the practice is expected to continue within certain cultural communities, while the prohibition may increase the risks for patients who are unable to provide proof of virginity or may seek alternative, clandestine practices.

Switzerland, on the other hand, has neither specific legislation nor professional guidelines addressing these issues. Consequently, there is no standardized approach among health professionals, and patient requests may be denied by some physicians but accepted by others. Moreover, Switzerland is a country where hymenoplasty, or so-called “re-virgination surgery,” is widely practiced, including for international clients.^21^

The aim of this study was to investigate the knowledge, attitudes, and practices of gynecologists and pediatricians in Switzerland regarding virginity testing, virginity certification, and hymenoplasty and to identify gaps in professional knowledge to inform future training initiatives and official expert recommendations for health-care providers.

## Materials and methods

We developed a self-administered survey for gynecologists and pediatricians in Switzerland. The survey was drafted in French, German, and Italian (the three national languages) and distributed via email through the national gynecologists’ and pediatricians’ societies. Due to a low response rate in the Italian-speaking region, a second invitation was extended by directly emailing the head physicians of gynecology and pediatric departments. Final-year medical students at the Geneva Medical Faculty also received the questionnaire via email from the faculty administration on two occasions, several months apart. We included this group because they are not yet practicing professionals, have no specific training or prior knowledge on the topic, and their participation allowed for a comparison between health-care professionals and a population without specialized expertise.

The survey consisted of 22 questions along with a free comment section and comprised of three sections: Knowledge, Attitudes, and Practices. It was available online on the Typeform platform from February to November 2022.

Data were extracted through Typeform and subsequently analyzed using R (version 4.2.2). To compare the proportions of different responses between groups, chi-square tests, or Fisher’s exact tests were performed where appropriate. Statistical significance was determined at a threshold of 5%. Items were categorized as follows: type of practice (medical practice vs. peripheral hospital vs. university hospital vs. private clinic, or public vs. private), specific training (having received coursework on these topics during medical school, residency, post-residency training, or never), working canton (German-speaking cantons vs. French-speaking cantons vs. Italian-speaking cantons), nationality (Swiss vs. non-Swiss), career stage (physicians vs. students, or gynecologists/obstetricians vs. pediatricians), age (as categorized in Table 1, or dichotomized as younger than 30 years vs. older than 30 years), and practice experience (health-care professionals who had received requests for virginity testing, virginity certification, or hymenoplasty vs. those who had not, and health-care professionals who had fulfilled such requests vs. those who had declined). Attitude items were classified into two categories: strongly agree/agree versus strongly disagree/disagree.

**Table 1:** General descriptive statistics.

**Variables**	**Total (*n* = 542)**
**Gender**	
Female	417 (76.9%)
Non-female (male and others)	125 (23.1%)
**Age (years)**	
<30	84 (15.7%)
30-40	162 (30.3%)
40-50	107 (20.0%)
40-60	114 (21.3%)
>60	68 (12.7%)
missing values	7
**Nationality**	
Switzerland	378 (69.7%)
Others	164 (30.3%)
**Career**	
Gyn/Obst (including residency)	371 (70.3%)
Pediatrician (including residency)	108 (20.5%)
Medical student	49 (9.3%)
missing values	14
**Job (more than one answer possible)**	
Medical practice	288 (53.1%)
Peripheral hospital	203 (37.5%)
University Hospital	132 (24.4%)
Private clinic	71 (13.1%)
**Working canton**	
German Switzerland	308 (57.7%)
French Switzerland	175 (32.8%)
Italian Switzerland	51 (9.6%)
missing values	8

## Results

A total of 542 responses were received. The results are summarized in Table 1.

### Knowledge

Nearly three-quarters of the participants (72.9%) were unaware of the official positions of scientific societies regarding virginity testing, virginity certification, and hymenoplasty. Answering this question was complex, as the Swiss Society of Obstetrics and Gynecologists (SGGG) does not have an official position on the topic. Most of the participants (82.3%) believed that an official position of the SGGG is important.

Approximately one-third of participants (29.7%) had never received coursework (or a component thereof) on the hymen, its anatomy, its function, and its impact on the sexual and psychological health of women. Of those who had received such training, 26.4% received it during residency, 29.5% received it during postgraduate and post-residency training, and 33.2% received it during medical school. When asked to rate the importance of such coursework in pre- and postgraduate curricula on a 0–10 scale, 18.5% chose 0–4 (no or little importance), 39.6% selected 5–7 (moderate importance), and 41.9% selected 8–10 (high importance).

### Attitudes toward virginity testing and related practices

Descriptive statistics from the portion of the survey on attitudes are reported in Table 2.

**Table 2:** Attitude descriptive statistics.

**Question**	**Strongly disagree + disagree**	**Strongly agree + agree**
Absence of bleeding during first vaginal penetration is a sign of non-virginity.	502 (97.5%)	13 (2.5%)
Hymenal rupture is the act that determines the lack of virginity.	433 (86.7%)	68 (13.3%)
The patient or a close relative has the right to request a virginity check.	412 (81.1%)	96 (18.9%)
Gynecologists can examine a patient to determine her virginity status.	386 (75.5%)	125 (24.5%)
Hymenal reconstruction is effective in ensuring bleeding during subsequent penetration in almost all cases.	420 (83.8%)	81 (16.2%)
Practicing hymenal reconstruction helps support patriarchy and reinforce gender inequalities.	73 (14.1%)	446 (85.9%)
A virginity certificate must be provided to the patient upon request, following an examination.	388 (76.8%)	117 (23.2%)
A virginity certificate must be provided to the patient only in life-threatening situations, including risk of death, social exclusion, or physical/psychological harm.	164 (32%)	348 (68%)

#### Beliefs about bleeding and virginity

Most of the participants (60%) strongly disagreed that the absence of bleeding during the first vaginal penetration indicates non-virginity; when “disagree” responses were included, this proportion increased to 97.5%. Thus, only 2.5% believed that bleeding is a sign of virginity. Despite this consensus, 13.3% of the participants endorsed the belief that hymenal rupture defines virginity loss. No significant associations were observed with any demographic or professional variables examined for these two items.

#### Feasibility of determining virginity status

While three-quarters of the participants (75.5%) disagreed or strongly disagreed that gynecologists can determine virginity status through examination, nearly one-quarter (24.5%) believed that this was possible. This latter belief was significantly associated with working in medical practice settings (*P* < .001), university hospitals (*P* = .01), private clinics (*P* = .009), and public institutions (*P* < .001). It was also more common among those lacking specific training, younger practitioners (particularly those under 30 years, *P* = .05), and those who had fulfilled patient requests for virginity testing (*P* < .001).

#### Beliefs about hymenal reconstruction

A minority of the participants (16.2%) agreed or strongly agreed that hymenal reconstruction ensures bleeding after subsequent penetration. Multivariate analysis revealed that this belief was significantly associated with having received specific training during residency or post-residency (*P* = .04), geographic location by canton (*P* = .01), younger age (*P* = .007), and having fulfilled patient requests for such procedures (*P* = .01).

In contrast, the overwhelming majority of the participants (85.9%) agreed or strongly agreed that performing hymenal reconstruction reinforces patriarchy and perpetuates gender inequalities. This view showed no significant variation across demographic or professional groups.

#### Acceptability of virginity testing

Approximately one-fifth of the participants (18.9%) agreed or strongly agreed that patients or their family members have the right to request virginity testing. This attitude was significantly more prevalent among those working in medical practices compared to other settings (22.5% vs. 14.9%, *P* = .03), varied by linguistic region (German-speaking 18.6%, French-speaking 15.5%, Italian-speaking 34.7%, *P* = .011), and was markedly higher among health-care professionals who had fulfilled such requests compared to those who had declined (28.2% vs. 11.5%, *P* < .001).

#### Provision of virginity certificates

Most participants (76.8%) disagreed or strongly disagreed with providing a virginity certificate at the patient’s request following an examination. This position was significantly associated with working in medical practice settings (*P* = .016), having received specific training during residency (*P* = .04), professional status (health-care professionals vs. students, *P* = .03), and age (*P* = .003), although the association was not significant for those younger than 30 years (*P* = .2). Those who had declined such requests were significantly more likely to oppose certification (*P* < .001).

However, when the scenario involved life-threatening circumstances, family or community exclusion, or physical or psychological violence, 68% of participants agreed or strongly agreed that a certificate should be provided. Support for certification in these circumstances was significantly higher among those not working in university hospitals (70.5%, *P* = .03), those in German-speaking cantons (73.9%, *P* = .001), those working in private practice settings (73.3%, *P* = .02), and those who had fulfilled such requests (*P* = .004).

### Clinical practice patterns

Among physician respondents, 39.3% reported having received at least one request for virginity testing, virginity certification, or hymenal reconstruction surgery. Such requests were significantly more common among gynecologists than pediatricians (*P* < .001). Of those who received requests, 30.4% fulfilled them. Notably, only 28.8% of physicians receiving requests offered patients a multidisciplinary management approach, including consultation with psychologists, anthropologists, or transcultural experts, or presented alternatives such as the use of artificial blood-colored products, physiotherapy, or other options prior to making a final decision. Multivariate analysis revealed no significant differences in the provision of multidisciplinary management approaches based on public versus private practice setting, physician gender, or age (Table 3).

**Table 3:** Physicians who proposed a multidisciplinary approach.

**Variables**	**No**	**Yes**	***P*-value**
	**(*n* = 173, 71.2%)**	**(*n* = 70, 28.8%)**	**(chi2)**
Working in public			.595
yes	80 (69.6%)	35 (30.4%)	
no	93 (72.7%)	35 (27.3%)	
Gender female			.994
yes	136 (71.2%)	55 (28.8%)	
no	37 (71.2%)	15 (28.8%)	
Age in years			.648^a^
<30	7 (63.6%)	4 (36.4%)	
30-40	49 (75.4%)	16 (24.6%)	
40-50	40 (71.4%)	16 (28.6%)	
40-60	45 (65.2%)	24 (34.8%)	
>60	31 (75.6%)	10 (24.4%)	
missing values	1		
Younger than 30 yo			.734^a^
yes	7 (63.6%)	4 (36.4%)	
no	165 (71.4%)	66 (28.6%)	
missing values	1		

^a^Fisher’s exact test.

## Discussion

While “virginity” is a social construct rather than a biological state, is not determined by any specific act, and is subject to variation across cultures, individuals, and sexual practices,^22^ for the purpose of this discussion, we operationally define “virgins” as women who have never experienced vaginal penetration.

Our study revealed that nearly 40% of relevant health professionals in Switzerland (gynecologists or pediatricians) have encountered requests for hymenoplasty, virginity examination, or virginity certification, and approximately one-third have fulfilled such requests. This occurs in the context of limited formal education on hymenal anatomy and function and targeted surgical training. Notably, 30% of survey respondents reported never having received any instruction on these topics throughout their medical careers. This underscores a critical knowledge gap with direct implications for clinical practice, particularly within a population of health-care professionals who are expected to possess the highest level of expertise in these domains.

According to our findings, 13.3% of respondents agreed (either agreed or strongly agreed) that hymenal rupture is crucial in determining a woman’s virginity. More concerningly, 24.5% of health professionals believed that they could examine a patient and determine their virginity status, with this belief being particularly prevalent among those working in a medical practice setting (32%, *P* < .001) and in private clinics (37.3%, *P* = .009). This proportion decreased to 15.9% among those working in university hospitals (*P* = .01). These misconceptions and practices can reinforce scientifically unfounded sociocultural beliefs and perpetuate a gendered double standard, as men are never required to prove their virginity or undergo “virginity reconstruction” before marriage. Also of concern, 14.1% of respondents did not agree that interventions promote gender inequality and patriarchy.

Consent to examination and surgery in these situations is questionable from a medical point of view but the implications are potentially dangerous for women who may not be able to prove their virginity otherwise.^1^ This concern likely explains why 23.2% of responders agreed to provide virginity certificates upon patient request following examination, a proportion that increased to 68% when life-threatening circumstances were involved or imminent. This willingness was significantly higher among physicians working in private clinics (73.3%, *P* = .024) or in German-speaking cantons (73.9%, *P* = .001). These findings are consistent with the debate in France where issuing such certificates is now illegal but where some French gynecologists continue to argue that they may be necessary to ensure patient safety.

Attitudes toward virginity examinations, virginity certificates, and hymenoplasty vary considerably between countries, and several studies have investigated these differences. A 2010 Swedish study revealed that, due to the absence of clear national guidelines at the time, case management varied depending on the health-care professional. Only 12% of respondents considered themselves sufficiently knowledgeable about the subject, and 5% reported refusing to provide care without referring the patient elsewhere.^23^ A subsequent Swedish study from 2013,and further confirmed in a another later study, noted similar conclusions of variation in care depending on the care provider.^24^^,^^25^ Recently, Sweden has adopted a formal position by banning virginity testing and virginity certificates, with legislation expected to take effect by the end of 2025.^26^ A lack of guidance and heterogeneity in accepting requests for hymenoplasty were also reported in a Belgian study in 2018.^27^ Virginity testing and hymenoplasty are banned in the UK, including when performed abroad, and are classified as forms of “honor-based” and domestic abuse.^28^^,^^29^

These findings have contributed to the ongoing debate about adopting a zero-tolerance approach. Providing services such as hymenoplasty or virginity examinations under certain circumstances may inadvertently reinforce the misconception that virginity is an anatomical condition rather than a social and personal construct. Despite the WHO’s clear position against virginity testing, current practices regarding virginity testing, virginity certification, and hymenoplasty remain heterogeneous and are not regulated by national or international scientific medical societies. While sexual and reproductive education is considered essential for eradicating false beliefs about virginity, some gynecologists openly support performing these procedures, citing reasons such as female empowerment, respect for autonomy, pragmatic decision-making, or the need to protect patients whose lives may be at risk,^30–32^ despite other potential risks and long-term consequences for sexual and psychological health.^6^

This debate parallels the ongoing discussion surrounding FGM. While there is a general zero-tolerance approach toward FGM among health-care professionals in Western countries, some researchers consider that cosmetic genital surgeries, such as hymenoplasty, are anatomically similar to and potentially as harmful as some forms of FGM and should, therefore, also be more strictly regulated from a legal, ethical, and medical point of view.^33^ A 2017 survey conducted in the United States investigated so-called “honor-related practices,” such as virginity testing, hymen reconstruction, and FGM, emphasizing that these practices can, in some respects, be considered within the same framework.^34^

Our multivariate analysis examined differences in attitudes according to the respondents’ ages. The results showed that younger physicians (less than 30 years old and 30–40 years old) were less likely to agree that hymen examination can determine virginity status (15.9% vs. 24.5%, *P* < .05). Conversely, older doctors (> 60 years old) were more likely to believe that hymenoplasty effectively induced bleeding after the first intercourse (29.5% vs. 16.2%, *P* = .007) and to support the provision of virginity certificates following examination (36.1% vs. 23.2%, *P* = .003). This is unsurprising, as younger generations have greater exposure to topics such as inclusivity and gender inequality through social media,^35^ while these topics are not mandatory components of the periodic professional development required throughout medical careers.

Multivariate analysis by canton yielded inconsistent results across attitudes, and we were unable to identify any clear trends, except for a greater propensity among physicians in German-speaking cantons to provide virginity certificates in life-threatening situations. This contrasted with French-speaking regions which may be reflective of the influence of the current debate.

We also found no consistent trends according to practice setting, except for specific attitudes such as the belief that gynecologists can determine virginity status through physical examination and, as reported earlier, a greater willingness to provide virginity certificates in private clinics or medical practices. Given the costs of hymenoplasty (up to 4000 CHF in Switzerland), it is plausible that private practitioners may respond positively to such requests primarily for financial reasons rather than for ideological ones.

Overall, among the survey respondents, physicians who actually fulfilled patient requests for virginity testing, virginity certification, or hymenoplasty were significantly more likely to believe that (1) patients have the right to make such requests, (2) physicians can determine virginity status, (3) hymenoplasty effectively induces bleeding, and (4) certificates should be provided upon request. This suggests that these beliefs are associated with actual practice in Switzerland.

An important question remains: How should health-care professionals respond to women requesting hymenoplasty, virginity testing, or virginity certification when they wish to decline the request while still protecting and supporting the patient? The decision faced by health-care professionals when responding to a request for hymenoplasty is ethically complex. On the one hand, patient autonomy must be respected: women have the right to make decisions regarding their own bodies, and hymenoplasty is generally classified as a cosmetic procedure. On the other hand, the principles of safety and non-maleficence are central to medical practice and although hymenoplasty involves surgical risks without clear medical indication, refusal may expose the patient to serious harm.^36^

Additionally, clinicians must uphold principles of non-discrimination, honesty, and integrity and may end up acting in ways that are inconsistent with their own beliefs.. Despite this complexity, requests for hymenoplasty remain largely overlooked in medical training, education, and the scientific literature.

Several alternatives to surgery exist, including perineal physiotherapy and artificial blood capsules tailored to the patient’s needs.^30^ However, only 28.8% of our respondents reported offering multidisciplinary management or non-surgical alternatives. Given the complexity of hymenoplasty and the potential for risk of life and harm, psychological and sex education support should always be provided regardless of whether surgery is performed. At present, alternative approaches are insufficiently explored in the scientific literature, resulting in limited training for health-care professionals and fewer options offered to patients.

It is important to note that comprehensive information and counseling can lead to the withdrawal of hymenoplasty requests in up to 75% of women, according to a Dutch study.^37^ In addition, in order to offer such counselling and support, health-care professionals must be properly trained and free from misconceptions about the hymen and virginity. Interestingly, in this particular study, only 2 of the 19 patients with follow-up reported bleeding after surgery. Given these data, it appears that alternatives to hymenoplasty may be more reliable than the surgery itself in inducing bleeding. Patient education—and, when appropriate, the education of family members and future husbands—is crucial, even though meaningful change requires time and sustained effort. Moreover, the responsibility for social change should not rest solely on women, nor should they bear the burden of potential consequences alone.^38^ Unlike France, Switzerland currently has no official position on this issue and no dedicated support network for patients and physicians, despite 82.3% of our survey participants considering an official statement from the SGGG important. Previous research has shown considerable variations in attitudes across Swiss clinics, with some performing hymenoplasty after only a brief interview and without multidisciplinary assessment or specialist consultation.^21^

### Strengths and limitations

Our study has several strengths and limitations. This is the first national-level survey on this topic and reflects Swiss knowledge, attitudes, and practices among gynecologists/obstetricians and pediatricians, the two specialties most frequently confronted with these issues. Despite the substantial response rate (542 responses, including 371 gynecologists/obstetricians), subgroup sizes were small. A larger study might elucidate additional differences according to age, canton, or practice setting.

The predominance of female respondents may be attributable to the gender distribution within the surveyed specialties, as Obstetrics and Gynecology and Pediatrics are known to have a high proportion of female practitioners.^39^ Another possible explanation is that female physicians may have a greater interest in, or awareness of, issues related to hymenoplasty, which could have influenced their likelihood of responding to the survey. Nevertheless, the present study did not assess factors associated with survey participation; therefore, any such interpretation should be considered speculative.

Furthermore, we believe that beyond age, training, knowledge, canton, or the other variables examined, personal attitudes and practices are likely influenced by individual beliefs, which our survey did not capture. Moreover, discrepancies may occur within the same provider, depending on individual circumstances, and may reflect conflicting or imprecise attitudes and beliefs. For example, the same provider may believe that such practices reinforce patriarchy while simultaneously seeking to protect their patients. Although our study was not designed to assess these variations, this may further highlight the need for improved training and education and clear official guidelines. We also acknowledge possible response bias, as individuals already trained and interested in the subject may have been more likely to respond.

## Conclusions

This national survey revealed significant knowledge gaps and practice variability among Swiss gynecologists and pediatricians regarding virginity testing, virginity certification, and hymenoplasty. While younger physicians demonstrated more evidence-based attitudes, the persistence of these scientifically unfounded practices perpetuates gender inequality and exposes women to harm. This knowledge gap is particularly concerning among those health-care professionals who are expected to possess the highest level of expertise in these domains.

Switzerland lacks official guidelines and coordinated support networks such as those present in neighboring countries. We recommend that Swiss medical societies establish clear, evidence-based recommendations prioritizing patient information, safety, and support. Comprehensive education on hymenal anatomy and the social construction of virginity must be integrated into medical curricula. Health-care institutions should develop multidisciplinary support systems to provide holistic care for women consulting for these pressures, and a national network should be created to assist health-care professionals in navigating these ethically complex situations. Addressing these issues requires both medical leadership and broader societal efforts to challenge harmful beliefs about virginity and gender.
